# A genome‐wide association study for recurrent laryngeal neuropathy in the Thoroughbred horse identifies a candidate gene that regulates myelin structure

**DOI:** 10.1111/evj.14461

**Published:** 2025-01-10

**Authors:** Charlotte L. McGivney, Beatrice A. McGivney, Gabriella Farries, Katie F. Gough, Haige Han, Amy R. Holtby, David E. MacHugh, Lisa Michelle Katz, Emmeline W. Hill

**Affiliations:** ^1^ UCD School of Agriculture and Food Science University College Dublin Dublin Ireland; ^2^ Plusvital Ltd., The Highline, Dun Laoghaire Industrial Estate Dublin Ireland; ^3^ UCD Conway Institute of Biomolecular and Biomedical Research University College Dublin Dublin Ireland; ^4^ UCD School of Veterinary Medicine University College Dublin Dublin Ireland

**Keywords:** *DAAM2*, genetics, horse, *LRFN2*, myelin, recurrent laryngeal neuropathy, risk loci, RLN, upper respiratory tract

## Abstract

**Background:**

Equine recurrent laryngeal neuropathy (RLN) is an economically important upper respiratory tract (URT) disease with a genetic contribution to risk, but genetic variants independent of height have not been identified for Thoroughbreds. The method of clinical assessment for RLN is critical to accurately phenotype groups for genetic studies.

**Objectives:**

To identify genetic risk loci for RLN in Thoroughbreds in a genome‐wide association study (GWAS) following high‐resolution phenotyping.

**Study design:**

Case–control.

**Methods:**

Thoroughbred horses were characterised as RLN cases and controls using resting and exercising URT endoscopic examinations and laryngeal ultrasonography, with the case‐cohort supplemented using a questionnaire. Genotypes for 43 831 autosomal single‐nucleotide polymorphisms (SNPs) from *n* = 235 horses (*n* = 110 cases; *n* = 125 controls) were used to estimate trait heritability and identify significantly associated SNPs in a GWAS. Haplotypes were examined in cases and controls and risk allele frequencies were examined in a population cohort (*n* = 3126).

**Results:**

Heritability was *h* ^2^ = 0.30 including sex and 5PCs as covariates. A SNP on ECA20 located between candidate genes, *DAAM2* and *LRFN2*, was significantly associated with RLN. Six index SNPs with allelic effect sizes OR = 1.5–2.9 were identified on ECA1, ECA14, and ECA20 close to candidate genes *ATPA10*, *KCNN2*, and *TFAP2A*. Eleven ECA20 SNPs defined seven haplotypes with homozygous H2/H2 horses having a 3.1× higher risk of RLN. Risk alleles segregate in the population, and stallions are carriers.

**Main limitations:**

The main study population was young. Horses in the control group had no evidence of RLN as 2‐ or 3‐year olds but may have developed RLN later.

**Conclusions:**

Genetic markers for RLN were identified which may be useful for the development of a polygenic risk score. Candidate genes with functions in neuropathies may further the understanding of RLN pathobiology.

## Resumen

**Historial:**

La neuropatía laríngea recurrente (RNL) en el equino es una enfermedad del tracto respiratorio superior (URT) económicamente importante con una contribución genética al riesgo, cuyas variantes genéticas independientes a la altura no han sido identificadas en el Fina Sangre de Carrera. El método de diagnóstico clínico de RNL es crítico para determinar grupos fenotípicos para estudios genéticos.

**Objetivos:**

Identificar loci de riesgo genético para RLN en Fina Sangre de Carrera en un estudio de asociación de genoma generalizado (GWAS) después de fenotipificación de alta resolución.

**Diseño del Estudio:**

Caso‐control.

**Métodos:**

Caballos Fina Sangre de Carrera fueron caracterizados como casos RNL y controles usando examinaciones en reposo y durante ejercicio por endoscopia del URT y ultrasonido de la laringe, con la cohorte de casos suplementados usando un cuestionario. Lo genotipos de 43 831 polimorfismos autosomales de nucleótido singular (SNPs) de *n* = 235 caballos (*n* = 110 casos; *n* = 125 controles) fueron usados para estimar la heredabilidad del rasgo e identificar SNPs asociados en forma significativa en un GWAS. Haplotipos fueron examinados en los casos y controles, y las frecuencias de los alelos de riesgo fueron examinadas en una cohorte de la población (*n* = 3126).

**Resultados:**

La heredabilidad fue de *h* ^*2*^ = 0.30 incluyendo sexo y 5PCs como covariantes. Un SNP en ECA20 localizado entre los genes candidatos *DAAM2* y *LRFN2,* fue asociado en forma significativa con la RLN. Seis SNPs índices con efecto alélico de tamaño OR = 1.5–2.9 fueron identificados en ECA1, ECA14 y ECA20 cerca de los genes candidatos *ATPA10*, *KCNN2*, y *TFAP2A.* Once SNPs en ECA20 definieron siete haplotipos en caballos homocigotos H2/H2 de tener un riesgo 3.1× mayor de RLN. Los alelos de riesgo son segregados en la población y los potros son portadores.

**Limitaciones Principales:**

La población principal era joven. Los caballos en el grupo control no tenía evidencia de RLN a los dos o tres años, pero pueden haber desarrollado RLN más tarde.

**Conclusiones:**

Se identificaron marcadores genéticos para RLN los cuales pueden ser útiles para desarrollar un puntaje de riesgo poligénico. Los genes candidatos con funciones en neuropatías podrían aumentar el conocimiento de la patobiología de RLN.

## 1 INTRODUCTION

Recurrent laryngeal neuropathy (RLN) has a reported prevalence of 15%–19% in Thoroughbreds.^1^, ^2^, ^3^, ^4^ Decreased abduction of primarily the left arytenoid cartilage of the larynx due to neurogenic atrophy of the laryngeal abductor muscle (*cricoarytenoideus dorsalis*, CAD) results in reduced airway diameter, increased inspiratory resistance, and increased work of breathing.^5^ Most research supports the disease to be a peripheral mononeuropathy of the recurrent laryngeal nerve, with a clear aetiology yet to be identified.^6^ RLN heritability has been estimated between 32%–46%,^7^, ^8^ supporting a strong genetic component to the disease.

Suggestive risk loci for RLN have been reported using equine single‐nucleotide polymorphism (SNP) genotyping arrays.^7^, ^9^ A family‐based multi‐breed study identified an 8 Mb region on ECA1 spanning *EGR2*.^9^ In a Thoroughbred cohort, a GWAS identified a significant association with a SNP upstream of *LCORL*,^7^ a gene associated with wither height.^10^, ^11^, ^12^, ^13^ Including height in the analysis removed the association, suggesting the SNP to be a marker for wither height rather than RLN. This is supported by several studies reporting height to be associated with RLN risk.^14^, ^15^, ^16^

Accurate phenotyping is important when investigating disease‐risk loci for complex diseases such as RLN, with clear differences between disease and control groups required for the identification of genetic differentiation. RLN is traditionally diagnosed via resting and/or exercising upper respiratory tract (URT) endoscopic examination.^3^ RLN is consistently diagnosed in horses during exercise as compared with resting examinations, with several studies reporting resting endoscopic assessment of laryngeal function to be variable and unreliable for prediction of exercising laryngeal function.^3^, ^17^, ^18^, ^19^ Furthermore, observer variability has been reported to be substantial‐to‐perfect for arytenoid symmetry grading at exercise as compared with poor agreement for resting grades.^20^ These results support exercising URT endoscopic examination (overground endoscopy, OGE) to be the most accurate and reliable way to clinically diagnose RLN. However, because RLN is believed to be progressive,^21^ the control group has historically been difficult to accurately define, especially when evaluating young horses. Ultrasonographic evaluation of the *cricoarytenoideus lateralis* (CAL) muscle has been reported to have a high sensitivity and specificity for RLN disease prediction. Changes in this muscle may occur before functional changes in arytenoid symmetry, supporting the use of this modality in combination with URT endoscopic examination to refine an RLN control phenotype.^22^

Accessing a suitably large sample for OGE is challenging. Therefore in this study, we supplemented a cohort of horses clinically diagnosed with RLN following high‐resolution phenotyping by OGE with horses assigned as cases on the basis of a retrospective questionnaire. The aim was to test the hypothesis that there are genetic risk loci for RLN in Thoroughbred horses.

## 2 MATERIALS AND METHODS

### 2.1 Study population

Resting and exercising OGE examinations were performed on healthy Thoroughbreds in one Flat‐race training yard under the same trainer between September 2012 and April 2016. Horses were included if they were fit and in full race training. All OGE examinations were performed during a sprint training session over a similar distance. Horses were excluded if they had undergone URT surgical intervention, if technical problems occurred, and/or if health and exercise conditions differed from the inclusion criteria. Age, sex, height, and pedigree were recorded for each horse.

The study sample set was a convenience sample, with access to a limited number of horses.

### 2.2 OGE instrumentation and exercise tests

Horses warmed up on a mechanical walker for 30–60 min before URT examination. A twitch was used to facilitate endoscope (Videomed GmbH) placement via the right nostril, positioned to give a clear view of the larynx. All horses were fitted with a heart rate (HR) monitor (Equine H7 heart rate sensor belt, Polar Electro) and global positioning device (GPS) (Viper, Statsports) or a mobile electrocardiogram (ECG)‐GPS device (Televet mobile, Engel Engineering Services GmbH) to record velocity, HR and exercise distance. Jugular venous blood was collected before (Lact0) and 5 min post‐exercise (Lact5) for plasma lactate measurement using an automated analyser (YSI 2300 stat plus, YSI UK Ltd).

Immediately after instrumentation, horses walked for 5–10 min before undergoing a low‐intensity warm‐up on an all‐weather racetrack (300 m walk, 900 m trot/slow canter, 100 m walk) followed by a 900 m high‐intensity gallop up a 2.7° incline. OGE video recording for all horses began after twitch removal and stopped when the horse re‐entered the yard. Three OGE examinations were carried out on turf (similar incline and distance to the all‐weather track), with *n* = 16 horses examined on a circular gallop. One horse was evaluated on a 10% inclined high‐speed equine treadmill (2000 m distance, 12.5 m/s peak velocity). All videos were blindly evaluated by one experienced viewer (ACVS board‐eligible [large animal] with ≥10 years specialist clinical experience) using VLC software (v2.0.5, VideoLAN; www.videolan.com). Arytenoid asymmetry was graded using the Havemeyer grading schemes.^23^

### 2.3 Laryngeal ultrasonography

Before OGE examination, laryngeal ultrasonographic images were captured for each horse using standard windows,^24^ and blindly analysed by one experienced observer. Subjective comparisons of echogenicity, muscle fibre alignment, and edge clarity of the right and left CAL muscles were performed (Figure S1). Sensitivity and specificity as compared with the OGE data were calculated before using the results to refine the phenotypes.

### 2.4 RLN case and control phenotype definition

Horses were categorised as RLN controls if they had equivalent left and right CAL muscle echogenicity, a resting endoscopic laryngeal grade ≤2.1, and an exercising endoscopic laryngeal Grade A. Horses were categorised as RLN cases if they had an abnormal left CAL muscle (hyperechoic/derangement in muscle fibre alignment/loss of edge clarity) and an exercising endoscopic laryngeal Grade B or C. Because of the likely progressive nature of RLN, there had to be complete agreement between the resting and exercising OGE examination and ultrasonography results for a horse to be categorised as a control. Horses with an exercising laryngeal Grade A were thus excluded if they had a resting laryngeal grade >2.1 and/or an abnormal left CAL muscle. To be categorised as a case, there had to be complete agreement between the exercising OGE examination and ultrasonographic results; this was to ensure the correct diagnosis of RLN. Thus, horses with an exercising laryngeal Grade B or C but an equivalent left and right CAL muscle echogenicity were excluded.

To increase the case sample size, a retrospective questionnaire was completed by the two most senior managers at the same Flat‐race training yard. Horses that were not part of the OGE cohort were included as cases if they were reported as having had surgery for RLN (‘Has this horse ever had surgery for RLN? Y/N/?’). The questionnaire was completed for 642 horses born between 2004 and 2015, of which 45 were recorded as cases. Sex and pedigree were recorded for each horse. Height data was not available for these horses.

A retrospective questionnaire was also completed by the manager of a set of horses with samples collected for commercial genetic testing. Nineteen horses recorded as having had surgery for RLN were included as cases. Sex was recorded for each horse. Height and pedigree data were not available for these horses.

### 2.5 DNA extraction, genotyping, and imputation

DNA was extracted from whole blood and genotyped using the Illumina EquineSNP70 Genotyping BeadChip or the Axiom Equine Genotyping Array (MNEC670, Affymetrix). Only individuals and SNPs with a genotyping rate >95% were included with a minor allele frequency threshold of >0.05 applied, resulting in a set of *n* = 43 831 autosomal SNPs common to the platforms. This SNP set was used for the heritability analysis as well as for the GWAS. SNPs that failed quality control were imputed using the software programme BEAGLE (version 3.3.2, https://faculty.washington.edu/browning/beagle/beagle.html).^25^ For 10 horses genotyped on the Illumina Equine SNP70 BeadChip and Axiom Equine Genotyping Array, post‐imputation concordance was >99%. For the horses that passed QC and were included in the GWAS, 44.3% (54.5% of cases, 35.2% of controls) and 56.7% (45.4% of cases, 64.8% of controls) were genotyped on the Axiom Equine Genotyping Array and the Illumina EquineSNP70 Genotyping BeadChip, respectively.

### 2.6 Data analysis

#### 2.6.1 Principal component analysis

Principal component analysis (PCA) was performed using the ‐pca 10 command in PLINK 1.9 (version 1.9, https://zzz.bwh.harvard.edu/plink/).^26^ Principal components (PCs) were plotted with points colour coded based on cases (blue) and controls (red), with the proportion of the variance explained by each PC given. Pruning the data set using the PLINK default settings ‐indep 50 5 2 resulted in an SNP set of 4239 SNPs. PCA plots using this SNP set were almost identical to the unpruned plots.

#### 2.6.2 Heritability

The proportion of phenotypic variance captured by the SNPs was calculated to derive heritability using genome‐wide complex trait analysis (GCTA).^27^ Heritability was calculated using a case–control binary approach, with age, sex, and height included as covariates for the OGE cohort, and for the full study set with sex and the first five PCs (5PCs) included as covariates. For the OGE cohort, genetic correlations between RLN and height were estimated using bivariate restricted maximum likelihood (REML) in GCTA.

#### 2.6.3 Genome‐wide association study

Genetic association analysis was performed using PLINK^28^ with sex and 5PCs included as covariates. The genome‐wide significance threshold was set at 1.14 × 10^−6^ accounting for independent effects from all SNPs. Considering the linkage disequilibrium (LD) structure in the population, LD pruning using the PLINK command ‐indep 50 5 2 resulted in 4239 independent SNPs, revising the genome‐wide significance threshold to 1.18 × 10^−5^.

Identification of genome‐wide significant SNPs can be challenging with small sample sizes and large numbers of SNPs, with many GWAS underpowered to detect SNPs with modest effects.^29^ Therefore, the clumping approach was used to identify highly correlated SNPs (‘clumps’) to define regions of interest containing index SNPs.^30^, ^31^ This method was performed using the default settings in PLINK to post‐process the GWAS results, with only the most significant SNP per region of the genome in linkage disequilibrium (LD) kept; only SNPs with *p* < 0.0001 were included in the clumping analyses. An LD threshold of *R* ^2^ > 0.5 and a physical distance threshold of 250 kb were selected when assigning SNPs to a clump.

#### 2.6.4 Haplotype analysis

Haploview was used to generate visualisations of the haplotype structure at the ECA20 locus using the 11 SNPs located between 39.5 Mb‐41.5 Mb (SNP IDs rs69172139, rs69172147, rs69172153, rs69172157, rs69172173, rs69172185, rs69172187, rs69172193, rs394369393, rs69173536, rs69173542). Haplotype maps were generated for all horses together and for cases and controls separately. Significant differences between the occurrence of the haplotypes in cases and controls were calculated using a chi‐squared test.

### 2.7 Population samples for allelic distribution analyses

The six index SNPs were extracted from archived genotypes for *n* = 3126 Thoroughbred horses sampled in Australia/New Zealand, Europe, and North America, including *n* = 233 stallions (sires), maintained in a commercial repository (Plusvital Ltd., Ireland). DNA extraction, genotyping, and quality control were performed as described for the original GWAS cohort. For the calculation of ECA20 haplotype frequencies in these cohorts, haplotype phasing was performed using Shapeit software.^32^

## 3 RESULTS

### 3.1 RLN case and control groups

**FIGURE 1:**
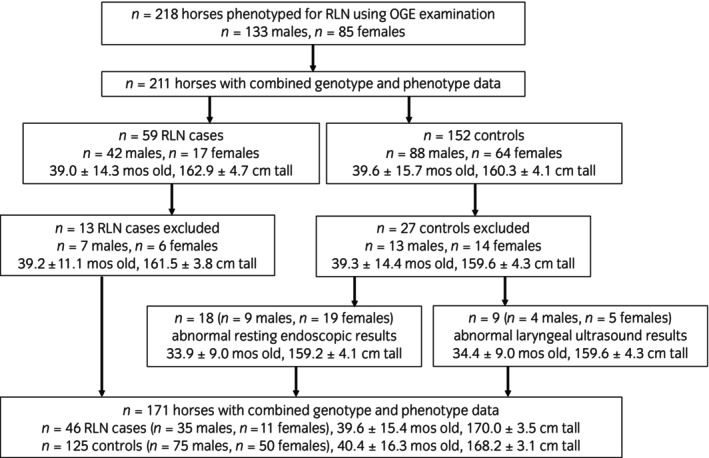
Flow chart detailing inclusion and exclusion criteria for cases and controls in the OGE cohort.

A total of *n* = 218 horses were phenotyped for RLN using exercising OGE examinations (Figure 1). Horses exercised a mean distance of 879.5 ± 265.7 m, achieving a mean peak velocity of 15.6 ± 1.6 m/s, a mean peak HR of 224.9 ± 7.2 bpm, and a mean peak Lact5 of 24.2 ± 6.0 mmol/L. Genotype data that passed standard quality control measures was generated for *n* = 211 phenotyped horses.

Comparison of laryngeal ultrasonographic measurements with exercising OGE results gave a sensitivity of 75%, a specificity of 93%, and an accuracy of 88%; this modality was thus used with resting endoscopic results to refine the groups. A total of *n* = 13 RLN cases and *n* = 27 controls were excluded (Figure 1). Cases were excluded due to disagreement between exercising OGE and laryngeal ultrasonographic results. Of the controls, *n* = 18 were excluded due to abnormal resting endoscopic examination results, and *n* = 9 due to abnormal laryngeal ultrasonographic findings; *n* = 1 had both abnormal resting endoscopic and laryngeal ultrasonographic results.

Therefore, *n* = 171 horses (*n* = 46 cases, *n* = 125 controls), with a mean age of 39.5 months, were included in the GWAS from the OGE study. Post hoc power calculations indicate that this sample size provides 78% power for an allelic effect size of 0.238 (OR = 2.91). Power calculation plots for the top three index SNPs in the GWAS (Table 1) are shown in Figure S2.

**TABLE 1:** Allele frequencies and genetic association results for index SNPs in the GWAS (*n* = 110 cases, *n* = 125 controls).

CHR	SNP	BP	A1	F_A	F_U	A2	CHISQ	*p* value	OR	SE	L95	U95
1	rs68618433	112 100 246	C	0.4091	0.192	T	26.59	2.52E−07	2.913	0.2112	1.926	4.407
14	rs69016935	57 668 633	G	0.3773	0.584	A	20.02	7.68E−06	0.4316	0.1892	0.2978	0.6253
20	rs69155142	10 819 751	A	0.2182	0.096	G	13.47	0.000243	2.628	0.2697	1.549	4.458
20	rs69173564	41 003 596	T	0.2273	0.12	G	9.534	0.002017	2.157	0.2525	1.315	3.538
20	rs69172139	40 502 757	C	0.3682	0.26	A	6.395	0.01144	1.659	0.2008	1.119	2.458
20	rs69172193	40 786 495	G	0.3545	0.268	A	4.109	0.04266	1.5	0.2006	1.013	2.223

Abbreviations: A1, Allele 1; A2, Allele 2; BP, base pair position of SNP; CHISQ, chi‐square statistic for the association; CHR, chromosome number; Freq A1 Case, frequency of allele 1 in cases; Freq A1 Control, frequency of allele 1 in controls; OR, odds ratio/allelic effect size; *p* value, allelic association with RLN, no covariates; SNP, SNP ID.

Height data was available for the OGE cohort, with cases taller than controls (*p* = 0.002). The mean height was 160.6 ± 4.0 cm for horses categorised as controls (exercising RLN Grade A) and 162.8 ± 4.7 cm for cases (exercising RLN Grades B/C) (Figure S3). When the cases were further separated, the mean height was 162.1 ± 4.2 cm for horses with exercising RLN Grade B and 165.0 ± 5.8 cm for horses with exercising RLN Grade C. An additional 64 cases were identified via a questionnaire resulting in a final GWAS sample set of *n* = 235 horses (*n* = 110 cases, *n* = 125 controls).

### 3.2 Principal component analysis

The first 5PCs explained 71.27% of the variation in the study population. There was no apparent clustering of cases separately from controls in the plots for PC1 (27.06%) versus PC2 (17.66%), PC1 versus PC3 (10.75%) with the cases evenly distributed among the controls (Figure S4). For the PC1 versus PC2 plot, there were three main clusters of horses, with the clusters predominantly the progeny of sires/related sire lines. The majority of the variation in the population was captured by the first 5PCs, with each of the additional PCs (PC6–10) providing marginal additional separation of the variance (5.29%–6.18%). Heights were visualised in PCA plots showing some clustering of tall cases (Figure S5).

### 3.3 Heritability estimates

For the OGE cohort the additive SNP heritability ($h_{\mathrm{SNP}}^{2}$) estimate for RLN was 0.41 (*p* = 3.4 × 10^−4^) (Table S1) with sex, age, and height included as covariates. Heritability was highest when only sex and age were included as covariates ($h_{\mathrm{SNP}}^{2}=0.44$; *p* = 3.51 × 10^−5^). While there was a significant difference in height between cases and controls (Figure S3), the genetic correlation between height and RLN was moderate (*r*_G_ = 0.31, Table S2). For the GWAS sample set, $h_{\mathrm{SNP}}^{2}$ for RLN was 0.30 (*p* = 6.6 × 10^−3^) (Table S1) with sex and 5PCs included as covariates.

### 3.4 Genome‐wide association study

**FIGURE 2:**
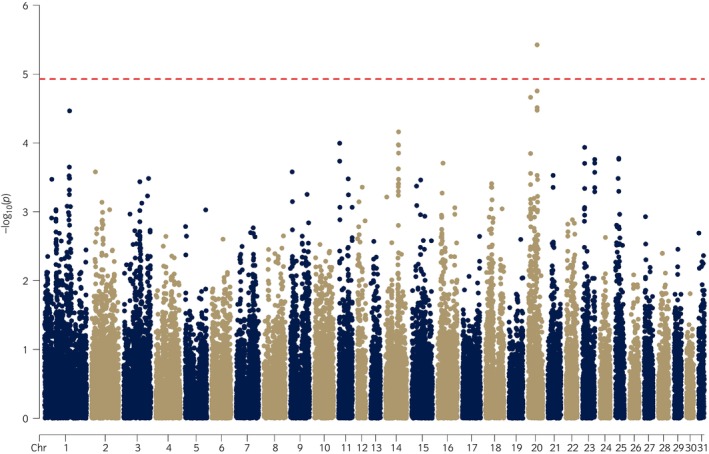
Manhattan plot for GWAS for horses phenotyped as RLN cases (*n* = 110) and controls (*n* = 125). Sex and 5PCs were included as covariates. A SNP on ECA20 was genome‐wide significant (*p* = 2.57 × 10^−6^).

A GWAS was performed for *n* = 235 horses (*n* = 110 cases, *n* = 125 controls) including sex and 5PCs as covariates. A SNP on ECA20 (rs69172139) reached genome‐wide significance (*p* = 3.75 × 10^−6^) (Figures 2 and S6; Table S3). Six of the top seven SNPs clustered in a 500 kb region on ECA20 (20: 40 502 757–41 003 596 bp). Applying the clumping method identified six index SNPs (*p* < 6.89 × 10^−5^), which included SNPs from the top ECA20 region, and three additional regions of interest on ECA1, ECA14 and ECA20, with the index SNPs (1: rs68618433, 14: rs69016935, 20: rs69155142) ranked 8th, 9th and 5th respectively in the GWAS (Table S4).

Allele and genotype frequencies were examined for the six index SNPs (Tables 1 and S5). For the top SNP on ECA20 (rs69172139) the C‐allele frequency was 36.8% in cases and 26% in controls (*p* = 0.01), with the CC genotype occurring in 10% of cases and 3.2% controls (*p* = 0.03). Six percent of horses were CC, with 73% being cases. For the SNP on ECA1 (rs68618433) the C‐allele frequency was 40.9% in cases and 19.2% in controls (*p* = 2.52 × 10^−7^), and the CC genotype occurred in 18.2% of cases and 2.4% controls (*p* = 4.9 × 10^−4^). Ten percent of horses were CC with 87% being cases, while 51% of horses were TT with 67% being controls.

### 3.5 Haplotype analysis

The haplotype structure at the ECA20 locus was examined using SNPs located between 39.5 and 41.5 Mb for all horses and cases and controls separately (Figure S7). Five of the top six ranked GWAS SNPs were within a single haplotype block (Block 3) which contained 11 SNPs (rs69172139, rs69172147, rs69172153, rs69172157, rs69172173, rs69172185, rs69172187, rs69172193, rs394369393, rs69173536, and rs69173542) located between 40.5 and 40.9 Mb. Seven haplotypes were defined using all horses. For cases, all SNPs were included in a single block (Block 5) with an additional SNP included (rs69172136). For controls, 5 SNPs were included in Block 5 with the others in a separate block (Block 6) with three additional SNPs included (rs69173543, rs69173564, and rs69173575).

The SNPs (rs69172139–rs69173542) defined four haplotypes (H1–H4) that occurred in 94.1% of cases and 82.2% of controls (Table 2). H1 was the most common (53.2% cases, 48.6% controls), H2 occurred in 35.5% of cases and 15.9% of controls (*p* = 6.0 × 10^−4^), H3 occurred in 3.6% of cases and 12.5% of controls (*p* = 0.012), and H4 occurred in 1.8% of cases and 5.2% of controls (*p* = 0.134). H2 was therefore considered a putative risk haplotype, with H3 a putative protective haplotype.

**TABLE 2:** Haplotypes (H1–H4) defined using the 11 SNPs identified on a single haplotype block on ECA20 with all horses included.

	26	27	28	29	30	31	32	33	34	35	36	Freq case	Freq control
H1	A	T	A	T	T	T	C	A	A	A	A	0.532	0.486
H2	C	C	G	C	C	C	T	G	G	G	G/A	0.355	0.159
H3	A	C	G	C	C	T	C	A	A	A	A	0.036	0.125
H4	A	C	G	C	C	T	C	A	G	A	G	0.018	0.052

*Note*: 26–36: SNP IDs rs69172139, rs69172147, rs69172153, rs69172157, rs69172173, rs69172185, rs69172187, rs69172193, rs394369393, rs69173536, rs69173542; Freq case, frequency of the haplotype in cases; Freq control, frequency of the haplotype in controls.

The occurrence of H1–H4 homozygotes and heterozygotes was then evaluated in the cases and controls (Table S6), combinations of which occurred in most of the horses (82.6%). H1/H1 (24.5% cases, 18.4% controls, *p* = 0.251) and H1/H2 (43.6% cases, 27.2% controls, *p* = 0.008) were the most common (56.2% of all horses). H1/H3 (4.5% cases, 8.8% controls, *p* = 0.196), H1/H4 (<1% cases, 5.6% controls, *p* = 0.048), and H2/H3 (2.7% cases, 8% controls, *p* = 0.078) occurred more frequently in controls, while H2/H2 occurred more frequently in cases (10% cases, 3.2% controls, *p* = 0.033). The H2 haplotype in the absence of H3 (i.e., H1/H2, H2/H2, H2/H4) occurred in 55% of cases and 30.4% controls (*p* = 1.4 × 10^−4^). This represents a 1.84× increased risk of RLN if a horse has at least one copy of H2 in the absence of H3, with H2/H2 horses having a 3.13× higher risk of RLN. This discrimination could be applied to 42.1% of the population containing these combinations of haplotypes.

### 3.6 Population risk‐allele and haplotype frequencies

The frequency of the risk alleles for each index SNP was examined in a stallion (sire) cohort (*n* = 233) and the general population (*n* = 3126) (Table S7, Figure S8). Five of the six risk alleles were at a lower frequency in the stallion cohort than in the cases, and four were at a lower frequency in the general population cohort than in the cases.

For the ECA20 haplotype, combinations of the H1–H4 haplotypes occurred in 43.9% of horses, compared with 82.9% of the study cohort (cases and controls). The H1/H2 haplotype occurred at a significantly lower frequency in the general population (*p* < 1.0 × 10^−5^) and in the sires (*p* = 2.2 × 10^−5^) than in the cases. There was no significant difference in frequency between cases and the general population cohort or the sire cohort for H2/H2 or H2/H4 (Table S6).

## 4 DISCUSSION

A considerable challenge to accurately identifying disease‐risk loci for complex traits is the definition of a robust, reproducible phenotype.^33^, ^34^ Here, a highly conservative inclusion/exclusion criteria for phenotyping RLN cases and controls were applied to the majority of the study cohort using resting and exercising arytenoid asymmetry and CAL muscle echogenicity. The control phenotype was the most stringently defined given that RLN appears to be progressive.^21^ Although it is possible that some horses in the control group may develop RLN as they age, among Thoroughbreds the risk of RLN onset is highest at 2–3 years of age.^15^, ^35^ The median age of the OGE cohort was 3.01 years, with the median age of retirement from racing among Flat racehorses reported to be 4.11 and 5.07 years for males and females, respectively.^36^ All horses were in active training and racing such that the genetic architecture underlying the measured phenotype would be relevant to the age at which the disease has the greatest economic effect in the Thoroughbred (Flat) racing ecosystem. Only controls defined by URT examinations were included since the high‐resolution phenotyping provided the best opportunity to exclude horses as cases. We considered that using a questionnaire to identify controls would be less robust since horses may have developed RLN without the knowledge of the respondents.

Heritability estimates were similar to previous reported measures.^7^ To maximise identification of the genetic contribution to the trait, the majority (92%) of the study horses were bred, managed, and trained at the same premises by one trainer, controlling for environmental variation as far as possible. Furthermore, the majority (70%) of the questionnaire case sample set was obtained from the same population of horses.

In the GWAS there was a significant hit on ECA20 at ~40.5 Mb. The top SNP was located between the *DAAM2* (dishevelled associated activator of morphogenesis 2; 20: 40 192 958–40 298 410 bp) and *LRFN2* (leucine‐rich repeat and fibronectin type III domain containing 2; 20: 40 696 011–40 924 384 bp) genes. Thereafter, many of the highest‐ranked ECA20 SNPs were located within *LRFN2*. In most cases, the causal gene contributing to disease is closest to the association signal.^37^, ^38^ Based on their functions in the nervous system, these two genes represent strong candidate genes for RLN.

*DAAM2* encodes a formin protein, which suppresses oligodendrocyte differentiation, is required to form the myelin sheath around axons, and is critical for the formation of functional myelin.^39^ During development, *DAAM2* is required for myelin compaction and in adults is necessary for motor learning. To form myelin, oligodendrocyte differentiation requires extensive alterations in cell morphology mediated by cytoskeletal remodelling proteins.^40^, ^41^ In demyelinating diseases such as Charcot–Marie–Tooth disease genetic variants in cytoskeleton‐associated genes have been implicated.^42^, ^43^ Charcot–Marie–Tooth disease is the most similar human pathology to equine RLN.^6^ It is as yet unclear whether the neuropathy developed with RLN is initiated at the myelin sheath or the axon; however, left‐sided demyelination and axonal loss is a hallmark of equine RLN.^44^ The identification of *DAAM2* as a candidate gene for RLN may aid in the functional classification of the disease.

*LRFN2*, also known as SALM1 (synaptic adhesion‐like molecule), is exclusively expressed in mouse embryonic neurons in the prospective hippocampus^45^ and controls synapse development by inducing neurexin clustering in hippocampal neurons.^46^ Synaptic adhesion molecules are known to regulate synapse development and function, controlling neural circuit and brain functions,^47^ and in *Lrfn2* ^−/−^ mice neurons displayed altered excitatory synaptic function.^48^ Behaviourally, the mutant mice had altered social communication and startle behaviours.

There were compelling candidate genes in the other regions of interest identified using the clumping method. The ECA1 SNP (rs68618433) was located within the *ATP10A* (ATPase phospholipid transporting 10A) gene, a member of the ATP gene family among which are genes associated with axonal degeneration.^49^ The ECA14 SNP (rs69016935) was <400 kb from the *KCNN2* (potassium calcium‐activated channel subfamily N member 2) gene encoding an integral membrane protein that forms voltage‐independent calcium activated channels. The other ECA20 region demarked by the rs69155142 SNP was 20 kb from the *TFAP2A* (transcription factor AP‐2 alpha) gene, a transcription factor that is involved in peripheral nerve repair and regeneration.^50^

As well as identifying candidate genes that may aid in understanding the pathophysiology of the disease, a key aim was to identify predictive genetic markers for the risk of developing RLN. We investigated whether any previously published markers for RLN in horses overlapped with the top 1% GWAS SNPs (Table S3). In Thoroughbreds, the *LCORL/NCAPG* locus on ECA3, which is associated with height in horses, was previously reported to be associated with RLN, but was not independent of height.^7^ Here an SNP (rs68599838) positioned 621 kb from *LCORL/NCAPG* was ranked 82nd in the GWAS. Although genome‐wide significance was not reached, the uncorrected *p*‐value (*p* = 0.00059) is highly significant if considered in terms of a single SNP replication analysis. In the OGE study cohort, horses with RLN Grade B/C tended to be taller (Figure S3), but the genetic correlation between height and RLN was moderate. In a mixed‐breed analysis, the *EGR2* locus on ECA1 was identified.^9^ Here, the closest SNP to *EGR2* ranked 114th in the GWAS. Finally, in American Belgian Draught horses a locus on ECA15 was identified to be independent of height,^51^ but in this study, the closest SNPs were >8 Mb from this locus. Therefore, other than for height, this study did not validate any previously published genetic markers for RLN. Furthermore, the markers identified in this study appear to be independent of height.

Among the SNPs identified in this study, examination of the index SNP allele and genotype frequencies and the ECA20 haplotype occurrence in cases and controls did not identify a single genetic marker that alone would provide high predictability for RLN in a suitably large proportion of the population that would be required for the development of a genetic screening/prediction test. However, this is not unexpected given the complex nature of the trait. Instead of relying on single genetic markers, polygenic risk scores (PRS) can be used to estimate individual genetic risk based on sets of SNPs determined from GWAS.^52^ For the six index SNPs, 10 controls had no risk alleles, whereas all cases had at least one risk allele, indicating that a PRS may be a promising approach. While PRS can be developed using SNPs that do not reach genome‐wide significance, index SNPs that are identified via clumping, combined with *p*‐value thresholds (clumping and threshold approach) are suggested to be optimal for PRS development.^52^

As for all scientific studies, to confirm the GWAS findings and the utility of a PRS for RLN risk, replication or validation analyses in an independent sample is important. However, accessing suitably large validation sets that are accurately phenotyped will be challenging, and may depend on cooperation from industry organisations and/or several participating veterinarians/trainers/breeding farms. In the absence of a validation sample, the index SNPs were examined in a large population cohort of horses representing the general population, and in a set of stallions. We expected each risk allele to occur at a higher frequency in cases and lower frequency in controls than in the general population, and we expected the risk alleles to be lower in the stallion cohort compared with the general population. For almost all the SNPs this pattern was observed, with risk alleles segregating in the population at frequencies of 0.23–0.39. While stallions are carriers, the incidence of the risk alleles was lower than in the general population (0.21–0.37). It is well known in the industry that there are stallions that more commonly produce horses with RLN and that these horses continue to be used for breeding. Judicial breeding using genetic tools could reduce the incidence of risk alleles in the population if stallion and mare genotypes are known. For the ECA20 haplotype, the H1/H2 genotype had significantly lower occurrence (0.06) in the general population than in the cases (0.44) but occurred at a frequency of 0.21 among the sires. This may reflect population genetic differences, as the general population included horses from geographic regions other than Europe, while the sires were principally European horses.

In conclusion, we have identified a set of SNPs that may be useful for the development of a PRS‐based screening test to assist in monitoring disease development, which may be beneficial for clinical management of at‐risk horses.^53^ Furthermore, integrating these SNPs in breeding decision‐making could lend toward the reduction of RLN incidence in the population by avoiding risk‐allele carrier matings. The identification of candidate genes with functions in nervous system development and neuropathies may also contribute to the understanding of RLN pathobiology in Thoroughbred horses.

## FUNDING INFORMATION

This study has emanated from research conducted with the financial support of Science Foundation Ireland (www.sfi.ie) under Grant Number 11/PI/1166.

## CONFLICT OF INTEREST STATEMENT

Zinto Labs (including Equinome, formerly Plusvital Ltd.) has been granted a licence from University College Dublin for exclusive commercial use of data reported in the manuscript. BAM, HH, and ARH are employed by Zinto Labs. EWH and DEM are shareholders in Zinto Labs.

## AUTHOR CONTRIBUTIONS

**Charlotte L. McGivney:** Investigation; writing – original draft; visualization; formal analysis. **Beatrice A. McGivney:** Investigation; methodology; writing – review and editing; formal analysis; validation. **Gabriella Farries:** Investigation. **Katie F. Gough:** Investigation. **Haige Han:** Formal analysis. **Amy R. Holtby:** Investigation. **David E. MacHugh:** Supervision. **Lisa Michelle Katz:** Conceptualization; investigation; writing – review and editing; methodology; validation; formal analysis; project administration; resources; supervision. **Emmeline W. Hill:** Conceptualization; investigation; funding acquisition; writing – original draft; writing – review and editing; methodology; project administration; supervision; resources.

## DATA INTEGRITY STATEMENT

Emmeline Hill had full access to all the data in the study and took responsibility for the integrity of the data and the accuracy of data analysis.

## ETHICAL ANIMAL RESEARCH

University College Dublin Animal Research Ethics Committee approval and an Irish Department of Health Licence (B100/3525) were obtained.

## INFORMED CONSENT

Owner/trainer informed consent was obtained for the use of the horses in the study. Samples from horses for the population‐based analysis were submitted to Plusvital Ltd. for commercial genetic testing. Explicit owner informed consent was not sought for this study specifically but the horse owners or their agents gave consent for data to be used for research in general.

### PEER REVIEW

The peer review history for this article is available at https://www.webofscience.com/api/gateway/wos/peer-review/10.1111/evj.14461.

## Supporting information


**Figure S1.** Diagnostic ultrasonographic images showing a longitudinal image of the left and right *cricoarytenoideus lateralis* muscles from a control horse (a and b) and an RLN case horse (c and d). In image c a region of echointensity and loss of muscle fibre definition is noted (>>>) within the *cricoarytenoideus lateralis*, typical of RLN and consistent histologically with fibrosis and fat infiltration, decreases in muscle fibre diameter, and a decrease in Type IIX muscle fibres.

**Figure S2.** Post hoc power calculation plots for the top three estimated allelic effect sizes for SNPs rs68618433, rs69016935, rs69155142 before (left), and after (right) LD pruning.

**Figure S3.** The relationship between height (cm) among control (RLN Grade A; blue) and case (RLN Grade B/C; orange) horses for *n* = 171 Thoroughbreds evaluated by overground endoscopy (Grade A, *n* = 126; Grade B, *n* = 37; Grade C, *n* = 9) (*p* = 0.002).

**Figure S4.** (a,b) Principal component analysis (PCA) plots based on genotype data for the horses used in the GWAS analyses. (a) PC1 vs. PC2, (b) PC1 vs. PC3; Individuals are colour coded on the basis of phenotype assignment; cases (red), controls (blue).

**Figure S5.** PCA plots with the size of the points relative to the height of the horse. (a) PC1 vs. PC2, (b) PC1 vs. PC3; Individuals are colour coded on the basis of phenotype assignment; cases (red), controls (blue).

**Figure S6.** Quantile–Quantile plot showing deviation from expected values for SNP association with RLN (*n* = 110 cases and *n* = 125 controls; sex, and 5PCs).

**Figure S7.** Haplotype maps for 11 SNPs (rs69172139, rs69172147, rs69172153, rs69172157, rs69172173, rs69172185, rs69172187, rs69172193, rs394369393, rs69173536, rs69173542) at the ECA20 locus containing the significant SNP in All, Cases, and Controls.

**Figure S8.** Frequency of risk alleles for the six index SNPs in cases, controls, stallions (sire), and in the general population (pop).

**Table S1.** Estimates of additive SNP heritability for RLN.

**Table S2.** Bivariate restricted maximum likelihood analysis of RLN case/control status and height, estimating the genetic variance of each trait and the genetic covariance between the two traits captured by all SNPs.

**Table S3.** Top 1% of SNPs in the GWAS for RLN including sex and 5 PCs as covariates.

**Table S4.** GWAS index SNPs ranked by *p*‐value for association with RLN following clumping.

**Table S5.** Allele and genotype frequencies for the six index SNPs.

**Table S6.** Occurrence of ECA20 haplotypes (H1–H4) in the homozygous or heterozygous state in cases and controls.

**Table S7.** Frequency of risk alleles for the six index SNPs in a stallion (Sire) cohort and in the general population (Pop).

## Data Availability

The data that support the findings of this study are available from the corresponding author upon reasonable request: Open sharing exemption granted by the editor due to lack of provision in the owner‐informed consent process.
