# Supplementary material for: A genome‐wide association study for recurrent laryngeal neuropathy in the Thoroughbred horse identifies a candidate gene that regulates myelin structure
Source: Equine Vet J. 2025 Jan 10;57(4):943–52. doi: 10.1111/evj.14461 (PMC12135753; doi:10.1111/evj.14461)

Equine Veterinary Journal BEVA

Figure S1: Diagnostic ultrasonographic images showing a longitudinal image of the left and right cricoarytenoideus lateralis muscles from a control horse (a and b) and an RLN case horse (c and d). In image c a region of echointensity and loss of muscle fibre definition is noted (>>>) within the cricoarytenoideus lateralis, typical of RLN and consistent histologically with fibrosis and fat infiltration, decreases in muscle fibre diameter and a decrease in Type IIX muscle fibres.

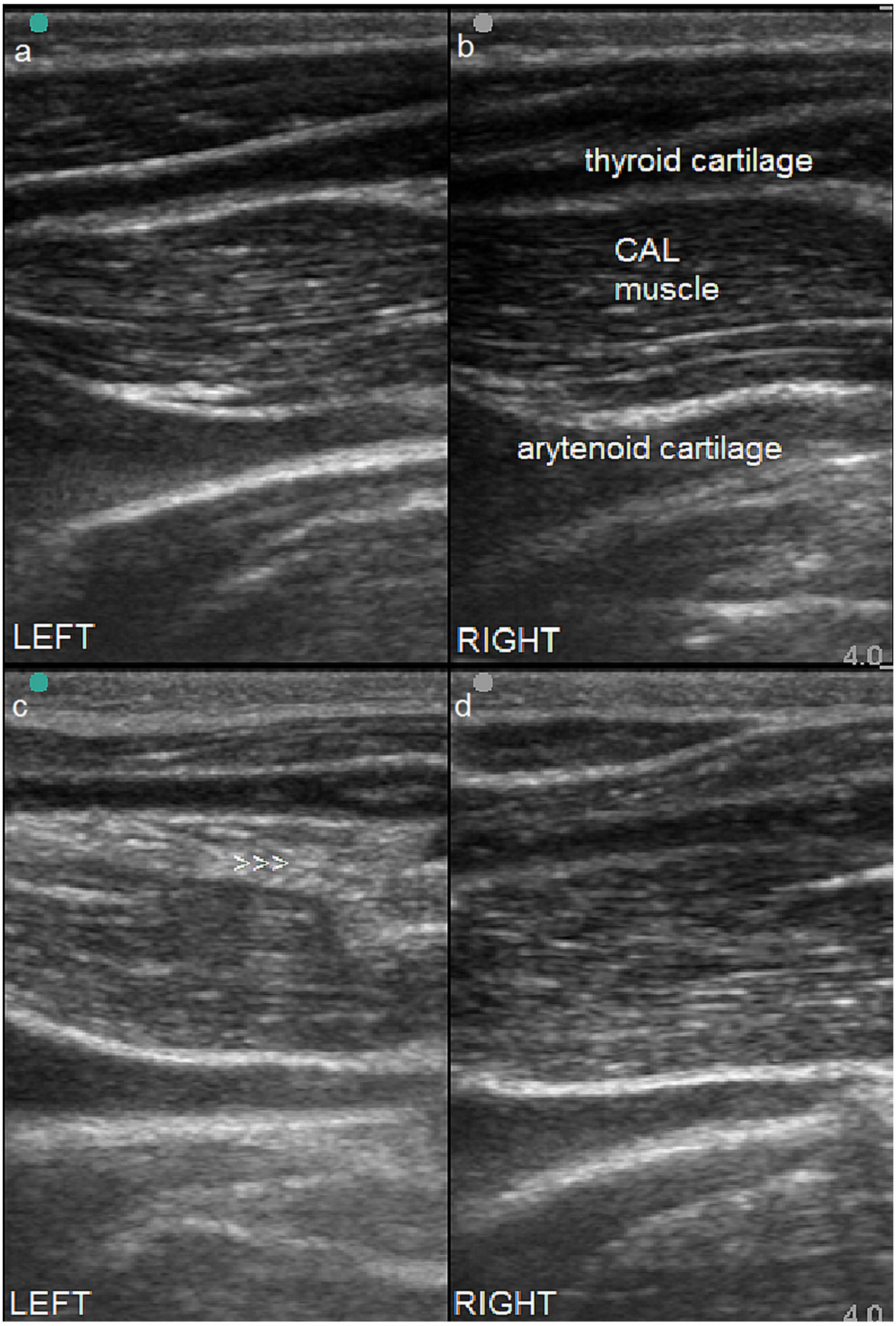

Supplement: Supplementary file 1 — Figure S1. Diagnostic ultrasonographic images showing a longitudinal image of the left and right cricoarytenoideus lateralis muscles from a control horse (a and b) and an RLN case horse (c and d). In image c a region of echointensity and loss of muscle fibre definition is noted (>>>) within the cricoarytenoideus lateralis, typical of RLN and consistent histologically with fibrosis and fat infiltration, decreases in muscle fibre diameter, and a decrease in Type IIX muscle fibres. [file EVJ-57-943-s013.pdf]
