# Supplementary material for: A genome‐wide association study for recurrent laryngeal neuropathy in the Thoroughbred horse identifies a candidate gene that regulates myelin structure
Source: Equine Vet J. 2025 Jan 10;57(4):943–52. doi: 10.1111/evj.14461 (PMC12135753; doi:10.1111/evj.14461)

Equine Veterinary Journal BEVA

Figure S2: Post-hoc power calculation plots for the top three estimated allelic effect sizes for SNPs rs68618433, rs69016935, rs69155142 before (left) and after (right) LD pruning.

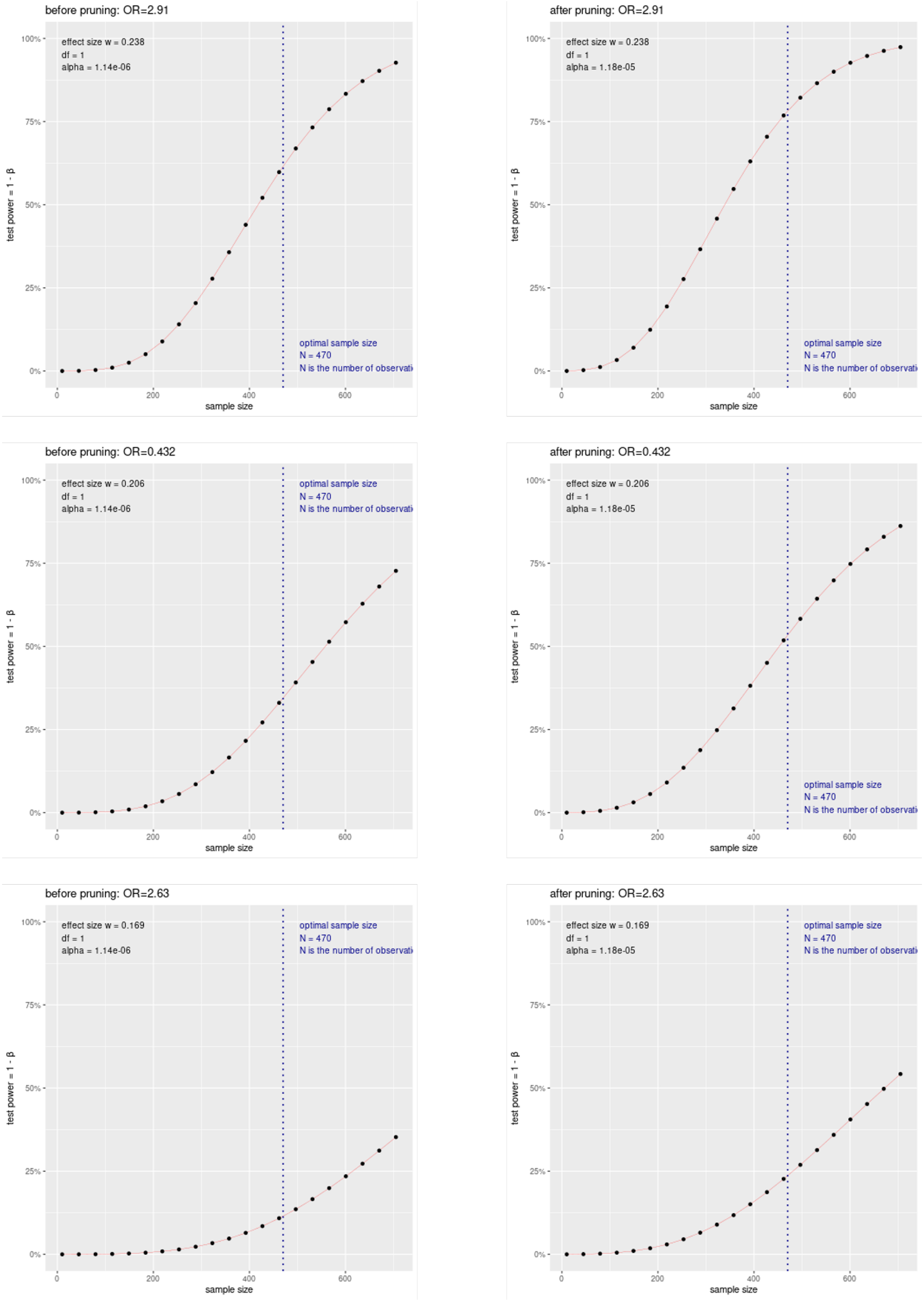

Supplement: Supplementary file 2 — Figure S2. Post hoc power calculation plots for the top three estimated allelic effect sizes for SNPs rs68618433, rs69016935, rs69155142 before (left), and after (right) LD pruning. [file EVJ-57-943-s001.pdf]
