# Supplementary material for: A genome‐wide association study for recurrent laryngeal neuropathy in the Thoroughbred horse identifies a candidate gene that regulates myelin structure
Source: Equine Vet J. 2025 Jan 10;57(4):943–52. doi: 10.1111/evj.14461 (PMC12135753; doi:10.1111/evj.14461)

Equine Veterinary Journal BEVA

Figure S3: The relationship between height (cm) among control (RLN grade A; blue) and case (RLN grade B/C; orange) horses for n=171 Thoroughbreds evaluated by overground endoscopy (Grade A, n=126; Grade B, n=37; Grade C, n=9) (P=0.002).

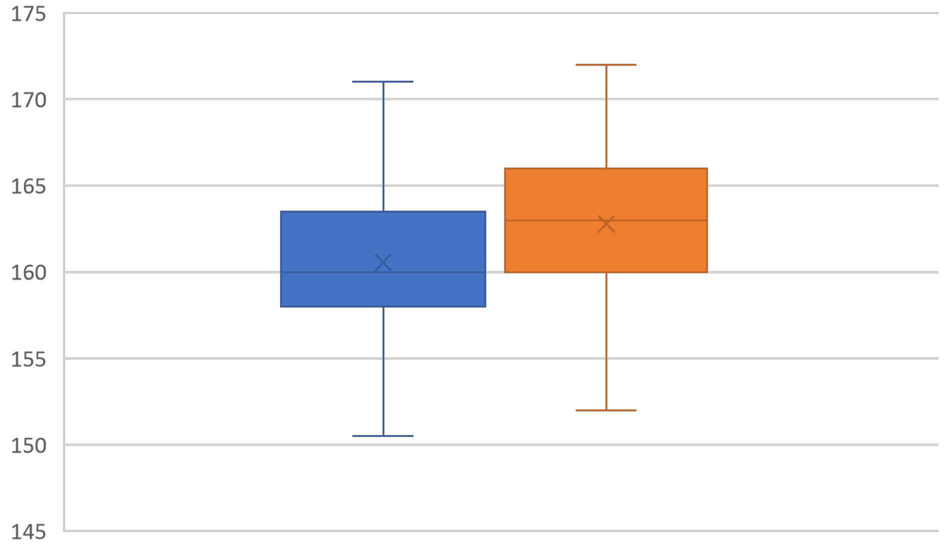

Supplement: Supplementary file 3 — Figure S3. The relationship between height (cm) among control (RLN Grade A; blue) and case (RLN Grade B/C; orange) horses for n = 171 Thoroughbreds evaluated by overground endoscopy (Grade A, n = 126; Grade B, n = 37; Grade C, n = 9) (p = 0.002). [file EVJ-57-943-s010.pdf]
