# Supplementary material for: A genome‐wide association study for recurrent laryngeal neuropathy in the Thoroughbred horse identifies a candidate gene that regulates myelin structure
Source: Equine Vet J. 2025 Jan 10;57(4):943–52. doi: 10.1111/evj.14461 (PMC12135753; doi:10.1111/evj.14461)

Equine Veterinary Journal
BEVA

Figure S5: Principal Component Analysis (PCA) plots with the size of the points relative to the height of the horse. a) PC1 vs PC2, b) PC1 vs PC3; Individuals are colour coded on the basis of phenotype assignment; cases (red), controls (blue).

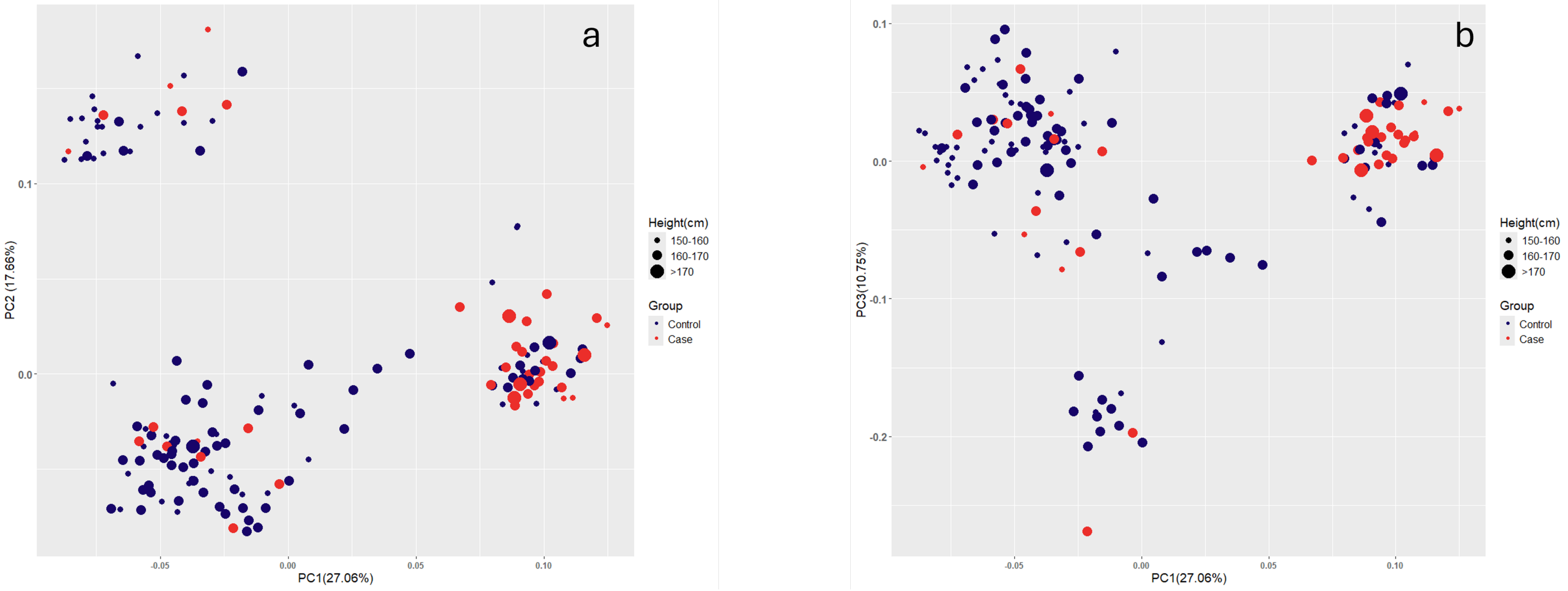

Supplement: Supplementary file 5 — Figure S5. PCA plots with the size of the points relative to the height of the horse. (a) PC1 vs. PC2, (b) PC1 vs. PC3; Individuals are colour coded on the basis of phenotype assignment; cases (red), controls (blue). [file EVJ-57-943-s012.pdf]
