# Supplementary material for: A genome‐wide association study for recurrent laryngeal neuropathy in the Thoroughbred horse identifies a candidate gene that regulates myelin structure
Source: Equine Vet J. 2025 Jan 10;57(4):943–52. doi: 10.1111/evj.14461 (PMC12135753; doi:10.1111/evj.14461)

Equine Veterinary Journal BEVA

Figure S6: Quantile-Quantile plot showing deviation from expected values for SNP association with RLN (n=110 cases and n=125 controls; sex, and 5PCs).

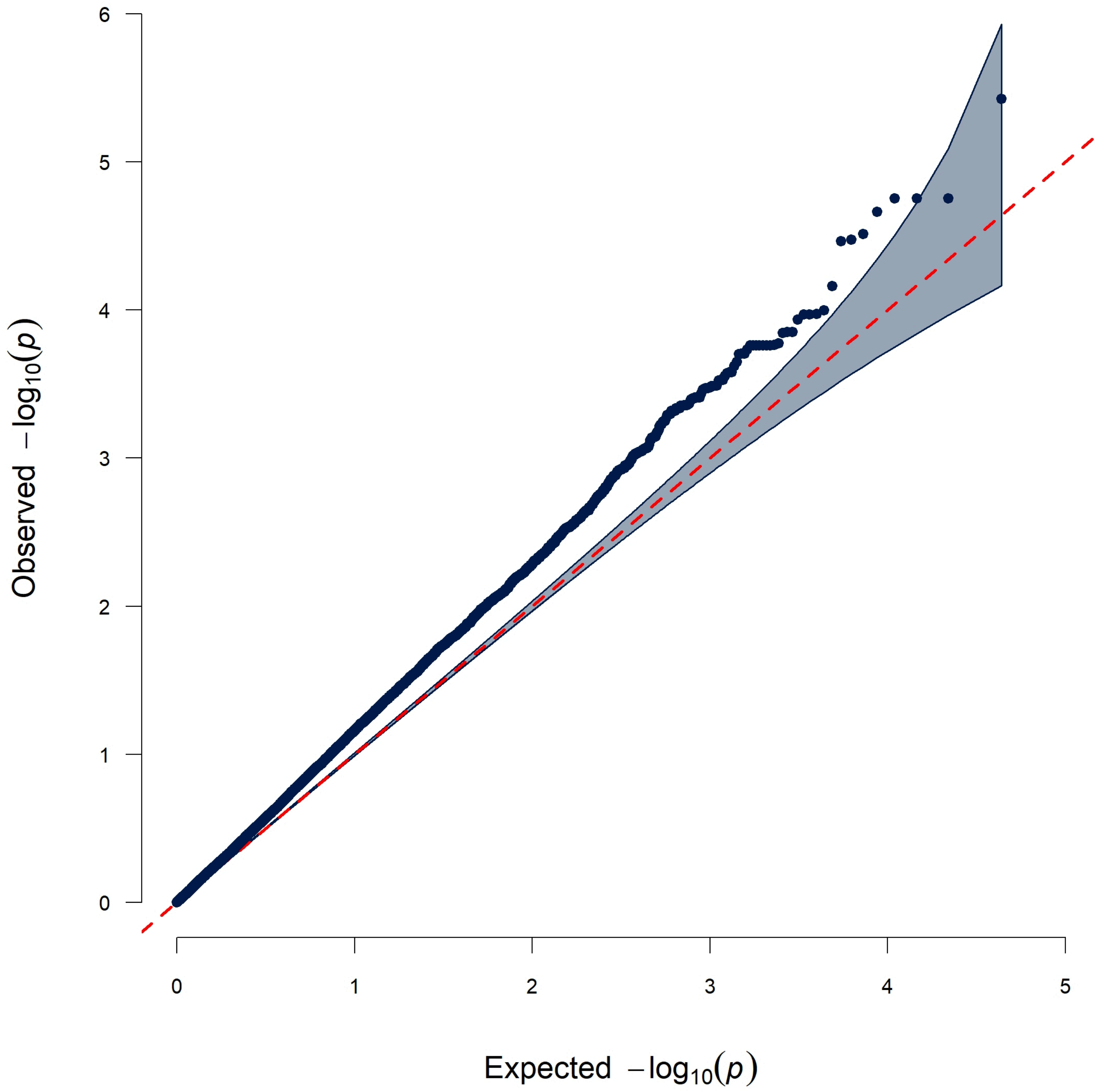

Supplement: Supplementary file 6 — Figure S6. Quantile–Quantile plot showing deviation from expected values for SNP association with RLN (n = 110 cases and n = 125 controls; sex, and 5PCs). [file EVJ-57-943-s014.pdf]
