# Supplementary material for: A genome‐wide association study for recurrent laryngeal neuropathy in the Thoroughbred horse identifies a candidate gene that regulates myelin structure
Source: Equine Vet J. 2025 Jan 10;57(4):943–52. doi: 10.1111/evj.14461 (PMC12135753; doi:10.1111/evj.14461)

Equine Veterinary Journal BEVA

Figure S7: Haplotype maps for 11 SNPs (rs69172139, rs69172147, rs69172153, rs69172157, rs69172173, rs69172185, rs69172187, rs69172193, rs394369393, rs69173536, rs69173542) at the ECA20 locus containing the significant SNP in All, Cases, and Controls.

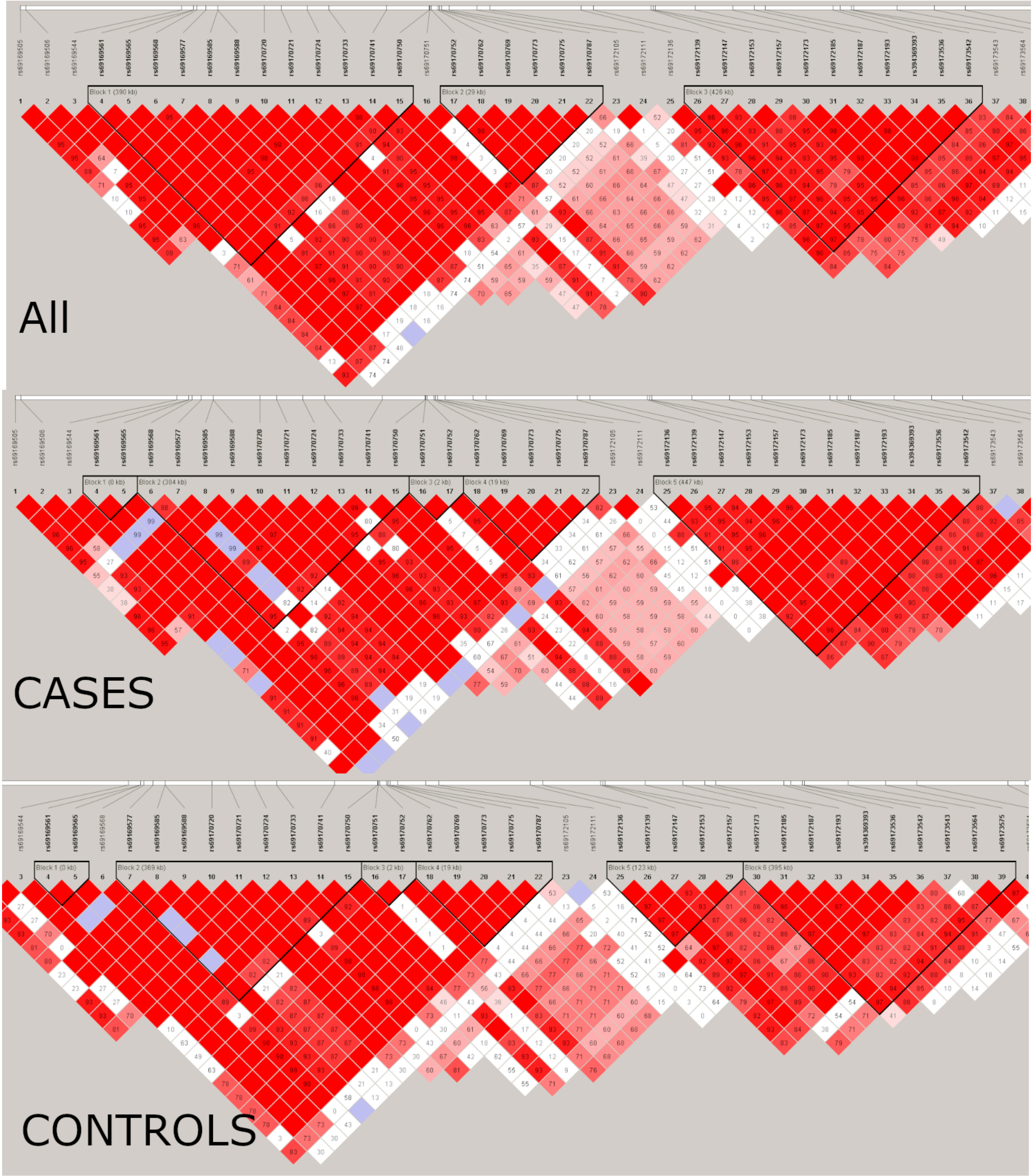

Supplement: Supplementary file 7 — Figure S7. Haplotype maps for 11 SNPs (rs69172139, rs69172147, rs69172153, rs69172157, rs69172173, rs69172185, rs69172187, rs69172193, rs394369393, rs69173536, rs69173542) at the ECA20 locus containing the significant SNP in All, Cases, and Controls. [file EVJ-57-943-s002.pdf]
