# Supplementary material for: A genome‐wide association study for recurrent laryngeal neuropathy in the Thoroughbred horse identifies a candidate gene that regulates myelin structure
Source: Equine Vet J. 2025 Jan 10;57(4):943–52. doi: 10.1111/evj.14461 (PMC12135753; doi:10.1111/evj.14461)

Equine Veterinary Journal BEVA

Figure S8: Frequency of risk alleles for the six index SNPs in cases, controls, stallions (sire) and in the general population (pop).

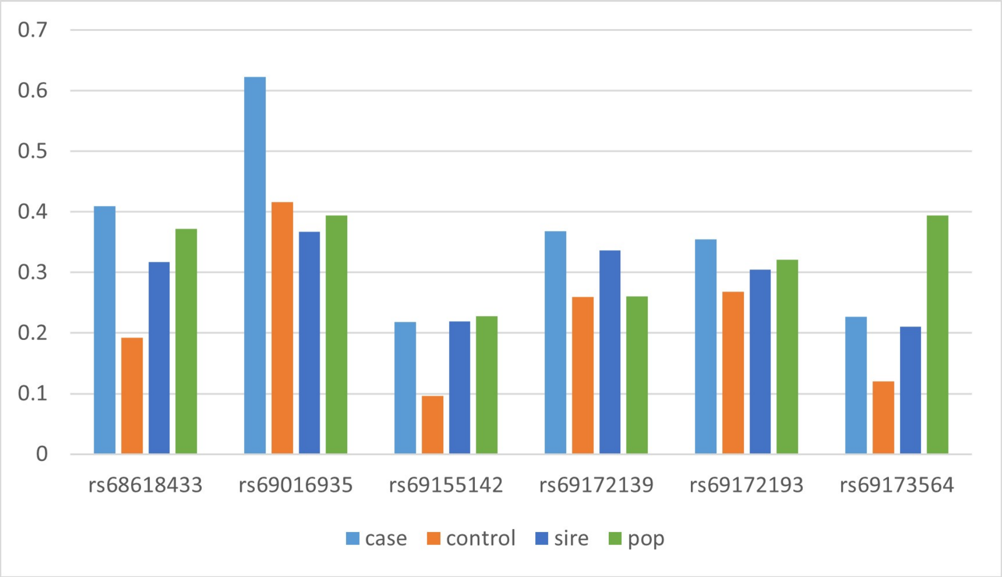

Supplement: Supplementary file 8 — Figure S8. Frequency of risk alleles for the six index SNPs in cases, controls, stallions (sire), and in the general population (pop). [file EVJ-57-943-s006.pdf]
