# Supplementary material for: A genome‐wide association study for recurrent laryngeal neuropathy in the Thoroughbred horse identifies a candidate gene that regulates myelin structure
Source: Equine Vet J. 2025 Jan 10;57(4):943–52. doi: 10.1111/evj.14461 (PMC12135753; doi:10.1111/evj.14461)
Supplement: Supplementary file 9 — Table S1. Estimates of additive SNP heritability for RLN. [file EVJ-57-943-s011.pdf]

Equine Veterinary Journal BEVA

Table S1: Estimates of additive SNP heritability for RLN. For the OGE cohort, covariates used were sex, age, and height; *n* = 171 Thoroughbred horses. For the GWAS cohort, covariates used were sex and first five principal components (5PCs); *n* = 235 Thoroughbred horses. (V(G) - Genetic variability; SE – Standard error; V(e) - Environmental variability; Vp - Total phenotypic variability; V(G)/Vp - Variability explained by SNPs; Pvalue - Significance under additive model)

**Covariates**	**V(G)**	**SE**	**V(e)**	**SE**	**Vp**	**SE**	**V(G)/Vp**	**SE**	***P* value**	**Cohort**
none	0.0822	0.0385	0.1075	0.0329	0.1897	0.0216	0.4331	0.1821	3.65E-05	OGE
sex	0.0825	0.0386	0.1056	0.0329	0.1881	0.0215	0.4384	0.1842	3.64E-05	OGE
age	0.0830	0.0388	0.1076	0.0331	0.1906	0.0218	0.4356	0.1824	3.55E-05	OGE
height	0.0733	0.0376	0.1103	0.0328	0.1836	0.0209	0.3995	0.1867	4.16E-04	OGE
sex, age	0.0834	0.0389	0.1053	0.0331	0.1887	0.0217	0.4419	0.1844	3.51E-05	OGE
sex, height	0.0748	0.0380	0.1089	0.0329	0.1837	0.0210	0.4071	0.1879	3.29E-04	OGE
age, height	0.0740	0.0380	0.1104	0.0330	0.1844	0.0211	0.4013	0.1873	4.31E-04	OGE
sex, age, height	0.0756	0.0383	0.1087	0.0331	0.1843	0.0212	0.4101	0.1885	3.35E-04	OGE
sex, 5PCs	0.0724	0.0349	0.1701	0.0304	0.243	0.0242	0.2985	0.1309	6.65E-03	GWAS
