# Supplementary material for: A genome‐wide association study for recurrent laryngeal neuropathy in the Thoroughbred horse identifies a candidate gene that regulates myelin structure
Source: Equine Vet J. 2025 Jan 10;57(4):943–52. doi: 10.1111/evj.14461 (PMC12135753; doi:10.1111/evj.14461)
Supplement: Supplementary file 10 — Table S2. Bivariate restricted maximum likelihood analysis of RLN case/control status and height, estimating the genetic variance of each trait and the genetic covariance between the two traits captured by all SNPs. [file EVJ-57-943-s008.pdf]

Table S2: Bivariate restricted maximum likelihood analysis of RLN case/control status and height, estimating the genetic variance of each trait and the genetic covariance between the two traits captured by all SNPs. (V(G)_tr1 - estimate of genetic variance for RLN; V(G)_tr2 - estimate of genetic variance for height; C(G)_tr12 - genetic correlation between RLN and height; V(e)_tr1 - residual variance for RLN; V(e)_tr2 - residual variance for height; (e)_tr12 - residual covariance between RLN and height; Vp_tr1 - proportion of phenotypic variance explained by all SNPs for RLN; Vp_tr2 - proportion of phenotypic variance explained by all SNPs for height; V(G)/Vp_tr1 - proportion of variance explained by all SNPs for RLN; V(G)/Vp_tr2 - proportion of variance explained by all SNPs for height; rG - genetic correlation).

V(G)_tr1	V(G)_tr2	C(G)_tr12	V(e)_tr1	V(e)_tr2	C(e)_tr12	Vp_tr1	Vp_tr2	V(G)/Vp_tr1	V(G)/Vp_tr2	rG
0.08	7.748	0.243	0.108	1.403	-0.025	0.188	9.151	0.426	0.847	0.308
