# Supplementary material for: A genome‐wide association study for recurrent laryngeal neuropathy in the Thoroughbred horse identifies a candidate gene that regulates myelin structure
Source: Equine Vet J. 2025 Jan 10;57(4):943–52. doi: 10.1111/evj.14461 (PMC12135753; doi:10.1111/evj.14461)
Supplement: Supplementary file 12 — Table S4. GWAS index SNPs ranked by p‐value for association with RLN following clumping. [file EVJ-57-943-s005.pdf]

Equine Veterinary Journal BEVA

Table S4: Index SNPs ranked by P-value for association with RLN following clumping, to identify regions of interest and SNPs for validation.

CHR	F	SNP	BP	P	TOTAL	NSIG	S05	S01	S001	S0001	SP2
20	1	rs69172139	40502757	3.75E-06	2	0	0	1	0	1	rs69172136(1),rs69172185(1)
20	1	rs69172193	40786495	1.76E-05	4	0	0	1	1	2	rs69172187(1),rs394369393(1),rs69173536(1),rs69173542(1)
20	1	rs69155142	10819751	2.17E-05	1	0	0	0	1	0	rs69153786(1)
20	1	rs69173564	41003596	3.33E-05	0	0	0	0	0	0	NONE
1	1	rs68618433	112100246	3.42E-05	2	0	0	1	1	0	rs68616995(1),rs68618487(1)
14	1	rs69016935	57668633	6.89E-05	6	0	0	2	4	0	rs69029597(1),rs69029600(1),rs69016909(1),rs69016925(1),rs69016929(1),rs69016934(1)
