# Supplementary material for: A genome‐wide association study for recurrent laryngeal neuropathy in the Thoroughbred horse identifies a candidate gene that regulates myelin structure
Source: Equine Vet J. 2025 Jan 10;57(4):943–52. doi: 10.1111/evj.14461 (PMC12135753; doi:10.1111/evj.14461)
Supplement: Supplementary file 13 — Table S5. Allele and genotype frequencies for the six index SNPs. [file EVJ-57-943-s007.pdf]

Equine Veterinary Journal BEVA

Table S5: Genotype occurrence for six index SNPs in cases and controls (A); frequency of each genotype in cases and controls (B); proportion of horses with the genotype as cases or controls (C).

A					B					C			
	rs68618433					rs68618433					rs68618433		
	TT	CT	CC	N		TT	CT	CC	N		TT	CT	CC
Case	40	50	20	110	Case	0.364	0.455	0.182	110	Case	0.333	0.543	0.870
Control	80	42	3	125	Control	0.640	0.336	0.024	125	Control	0.667	0.457	0.130
Total	120	92	23	235						N	120	92	23
	rs69016935					rs69016935					rs69016935		
	AA	GA	GG	N		AA	GA	GG	N		AA	GA	GG
Case	41	55	14	110	Case	0.373	0.500	0.127	110	Case	0.707	0.440	0.269
Control	17	70	38	125	Control	0.136	0.560	0.304	125	Control	0.293	0.560	0.731
Total	58	125	52	235						N	58	125	52
	rs69155142					rs69155142					rs69155142		
	GG	AG	AA	N		GG	AG	AA	N		GG	AG	AA
Case	67	38	5	110	Case	0.609	0.345	0.045	110	Case	0.399	0.613	1.000
Control	101	24	0	125	Control	0.808	0.192	0.000	125	Control	0.601	0.387	0.000
Total	168	62	5	235						N	168	62	5
	rs69172139					rs69172139					rs69172139		
	AA	CA	CC	N		AA	CA	CC	N		AA	CA	CC
Case	40	59	11	110	Case	0.364	0.536	0.100	110	Case	0.385	0.509	0.733
Control	64	57	4	125	Control	0.512	0.456	0.032	125	Control	0.615	0.491	0.267

**Total**	104	116	15	235						**N**	104	116	15
	**rs69172193**					**rs69172193**					**rs69172193**		
	**AA**	**GA**	**GG**	**N**		**AA**	**GA**	**GG**	**N**		**AA**	**GA**	**GG**
**Case**	43	56	11	110	**Case**	0.391	0.509	0.100	110	**Case**	0.406	0.496	0.688
**Control**	63	57	5	125	**Control**	0.504	0.456	0.040	125	**Control**	0.594	0.504	0.313
**Total**	106	113	16	235						**N**	106	113	16
	**rs69173564**					**rs69173564**					**rs69173564**		
	**GG**	**TG**	**TT**	**N**		**GG**	**TG**	**TT**	**N**		**GG**	**TG**	**TT**
**Case**	64	42	4	110	**Case**	0.582	0.382	0.036	110	**Case**	0.400	0.600	0.800
**Control**	96	28	1	125	**Control**	0.768	0.224	0.008	125	**Control**	0.600	0.400	0.200
**Total**	160	70	5	235						**N**	160	70	5
