# Supplementary material for: A genome‐wide association study for recurrent laryngeal neuropathy in the Thoroughbred horse identifies a candidate gene that regulates myelin structure
Source: Equine Vet J. 2025 Jan 10;57(4):943–52. doi: 10.1111/evj.14461 (PMC12135753; doi:10.1111/evj.14461)
Supplement: Supplementary file 14 — Table S6. Occurrence of ECA20 haplotypes (H1–H4) in the homozygous or heterozygous state in cases and controls. [file EVJ-57-943-s009.pdf]

Equine Veterinary Journal BEVA

Table S6: Occurrence of ECA20 haplotypes (H1 – H4) in the homozygous or heterozygous state in cases, controls, sire cohort, and general population cohort.

ECA20 haplotype	Freq Case (n=110)	Freq Control (n=125)	Freq Sire (n=233)	Freq Population (n=3126)
H1/H1	0.245	0.184	0.227	0.174
H1/H2	0.436	0.272	0.215	0.056
H1/H3	0.045	0.088	0.060	0.023
H1/H4	0.009	0.056	0.013	0.015
H2/H2	0.100	0.032	0.107	0.110
H2/H3	0.027	0.080	0.052	0.020
H2/H4	0.018	0.000	0.030	0.022
H3/H3	0.000	0.032	0.004	0.006
H3/H4	0.000	0.032	0.009	0.008
H4/H4	0.000	0.000	0.000	0.004
