# Supplementary material for: A genome‐wide association study for recurrent laryngeal neuropathy in the Thoroughbred horse identifies a candidate gene that regulates myelin structure
Source: Equine Vet J. 2025 Jan 10;57(4):943–52. doi: 10.1111/evj.14461 (PMC12135753; doi:10.1111/evj.14461)
Supplement: Supplementary file 15 — Table S7. Frequency of risk alleles for the six index SNPs in a stallion (Sire) cohort and in the general population (Pop). [file EVJ-57-943-s015.pdf]

Equine Veterinary Journal BEVA

Table S7: Frequency of risk alleles for the six index SNPs in a stallion (Sire) cohort and in the general population (Pop).

		Case	Control	Sire	Pop
**SNP**	**Risk allele**	**n=110**	**n=125**	**n=233**	**n=3126**
rs68618433	C	0.409	0.192	0.318	0.372
rs69016935	A	0.623	0.416	0.367	0.394
rs69155142	A	0.218	0.096	0.219	0.228
rs69172139	C	0.368	0.260	0.337	0.261
rs69172193	G	0.355	0.268	0.305	0.321
rs69173564	T	0.227	0.120	0.210	0.394
